# WONOEP XVII appraisal: The immunopathogenesis of epilepsy

**DOI:** 10.1111/epi.70027

**Published:** 2025-11-22

**Authors:** Nihan Çarçak, Said Al Maawal, Peravina Thergarajan, Tracie H. L. Tan, Vinod Kumar Mehta, Tamara Baker, David C. Henshall, Aristea S. Galanopoulou, Özlem Akman, Pablo M. Casillas‐Espinosa, Raman Sankar, Laura Librizzi, Adam E. Handel

**Affiliations:** ^1^ Department of Pharmacology, Faculty of Pharmacy Istanbul University Istanbul Turkey; ^2^ Institute of Health Sciences, Department of Neuroscience Acibadem Mehmet Ali Aydinlar University Istanbul Turkey; ^3^ Department of Neurology Alfred Health Melbourne Victoria Australia; ^4^ Department of Neuroscience, School of Translational Medicine Monash University Melbourne Victoria Australia; ^5^ Department of Neurology Geetanjali Medical University Udaipur India; ^6^ Department of Physiology and Medical Physics and FutureNeuro Research Ireland Centre RCSI University of Medicine and Health Sciences Dublin Ireland; ^7^ Albert Einstein College of Medicine Bronx New York USA; ^8^ Department of Physiology, Faculty of Medicine Demiroglu Bilim University Istanbul Turkey; ^9^ Department of Medicine, Royal Melbourne Hospital University of Melbourne Parkville Victoria Australia; ^10^ Department of Neurology and Pediatrics David Geffen School of Medicine at UCLA Los Angeles California USA; ^11^ Department of Clinical and Experimental Epileptology Fondazione IRCCS Istituto Neurologico Carlo Besta Milan Italy; ^12^ Nuffield Department of Clinical Neurosciences University of Oxford Oxford UK; ^13^ Department of Neurology John Radcliffe Hospital, Oxford University Hospitals Oxford UK

**Keywords:** autoimmunity, epilepsy, in vivo models, neuroinflammation, patient‐derived autoantibodies, seizures

## Abstract

There is a wealth of data indicating that the immune system plays an important role in seizure disorders. This includes autoimmune encephalitis, in which an immune response directed against neuronal antigens results in brain inflammation and subsequent seizure activity, as well as autoimmune‐associated epilepsy and neuroinflammatory changes detected in the tissues of patients with epilepsy, in which context it is often difficult to distinguish whether the inflammation is causal or a consequence of the seizures. Here, we summarize the discussion on this topic held during the XVII Workshop on Neurobiology of Epilepsy (WONOEP XVII), organized in 2023 by the Neurobiology Commission of the International League Against Epilepsy on the topic of the extracellular space in epilepsy. This critical appraisal explores the progress, emerging concepts, and discussion on the immunopathogenesis of epilepsy, with a particular focus on the interplay between the immune system and the central nervous system, highlighting the role of autoimmunity, neuroinflammation, and neuroimmunological processes in the etiology of various epileptic disorders and the prospects for new therapies.

**Key points**

- The mechanisms responsible for the development of epilepsies and the generation of spontaneous recurrent seizures are multifactorial.
- Innate and adaptive immune mechanisms underly ictal events.
- Immune responses require the collaboration of a broad spectrum of inflammatory systems, which may influence central nervous system activity and function.

## 1 INTRODUCTION

There is an increasing amount of evidence indicating dysfunction of the immune system in seizures and epilepsy disorders.^1^, ^2^, ^3^ This can conceptually be broken down into three main categories: (1) acute symptomatic seizures in the context of autoimmune encephalitis (AE), (2) autoimmune‐associated epilepsy, and (3) neuroinflammatory changes associated with epilepsy. All three areas were discussed during the XVII Workshop on Neurobiology of Epilepsy (WONOEP XVII), organized in 2023 by the Neurobiology Commission of the International League Against Epilepsy (ILAE), which focused on the extracellular space in epilepsy: pathomechanisms, biomarkers, and treatment targets. A key focus of the discussion of this topic was around the causal nature of the immune responses in each of these categories. This becomes progressively less clear, from seizures in the context of AE, in which an immune response directed against neuronal antigens results in brain inflammation and subsequent seizure activity, to neuroinflammatory changes detected in the tissues of patients with epilepsy, in which context it is often difficult to distinguish whether the inflammation is cause or a consequence of the seizures.^4^ This distinction is of clear clinical relevance, because intervening in a neuroinflammatory process has the potential to offer novel therapeutic mechanisms to control otherwise treatment‐refractory seizures. Conversely, this approach could cause considerable harm if immunotherapy is given inappropriately to patients. Here, we critically review current progress in this field and prospects for biomarkers and targeted therapies and identify knowledge gaps that could be closed by research in the future.

## 2 ACUTE SYMPTOMATIC SEIZURES IN THE CONTEXT OF AE

### 2.1 Introduction to AE

The term AE describes a group of conditions in which the immune system reacts against a target antigen within the brain to cause inflammation. AE manifests clinically as subacute cognitive, behavioral, and psychiatric symptoms along with other neurological manifestations, including seizures.^5^ Some of these seizure types can be important clues to the underlying immune response, such as the faciobrachial dystonic seizures seen in leucine‐rich glioma‐inactivated 1 (LGI1) antibody encephalitis.^6^ The immunopathogenesis of AE has been discussed elsewhere,^7^, ^8^ and the role of autoantibodies in driving ictal activity is discussed below. However, of particular relevance to this review is the association between the location of the target antigen either on the neuronal cell surface (e.g., N‐methyl‐d‐aspartate receptor [NMDAR] and LGI1) or intracellularly (e.g., glutamic acid decarboxylase [GAD] and Hu antigen) and the response to immunotherapy, which is typically good for cases associated with surface‐directed antibodies and poor for intracellular antigenic targets.^9^

### 2.2 Risk of postencephalitic epilepsy

Overall, most patients with AE do not suffer with epilepsy in the long term. Estimates of the risk of epilepsy following successful treatment of AE vary widely, although recent studies suggest that the overall risk may be as high as 29.2%.^10^, ^11^, ^12^, ^13^ Much of this disparity may be due to the differential risk of epilepsy with different antigenic targets. The type and frequency of seizures can be associated with prognosis in AE. For example, GAD antibody‐associated seizures are rarely responsive to treatment,^14^ and a high frequency of subclinical seizures is a strong predictor of poor outcome in LGI1 antibody encephalitis.^15^ Risk factors for persistent seizures likewise differ between studies but include hippocampal atrophy, status epilepticus, interictal epileptiform discharges, focal temporal lobe discharges, periodic discharges, and delayed immunotherapy.^11^, ^12^, ^13^ The risk of long‐term epilepsy differs by AE type, with surface‐directed neuronal antibodies less likely to lead to seizure persistence.^13^, ^16^ For example, the risk of postencephalitic epilepsy in LGI1 antibody encephalitis is 5.9%, far lower than the overall risk and with seizure remission occurring >2 years after disease onset in some patients.^17^

### 2.3 Treatment of seizures in AE

It is rare for seizures in the context of AE to respond to antiseizure medications alone.^10^ Immunotherapy is much more effective, and most patients with neuronal surface‐directed antibodies become seizure‐free.^13^, ^16^ A randomized controlled trial of intravenous immunoglobulin treatment showed that this could reduce seizure frequency in LGI1 or contactin‐associated protein‐like 2 (CASPR2) antibody encephalitis.^18^ Conversely, GAD antibody‐associated seizures rarely respond to immunotherapy, but there is evidence from a small trial that these may respond to combination treatment with clobazam and cenobamate.^14^, ^19^, ^20^ Thus, the frequency of postencephalitic epilepsy differs by antigenic target, but many types of AE respond to immunotherapy. The extent to which epilepsy associated with autoantibodies but without overt encephalitis is a distinct entity or exists on a continuum with AE is an important translational question.

## 3 INTRODUCTION TO AUTOIMMUNE‐ASSOCIATED EPILEPSY

**FIGURE 1:**
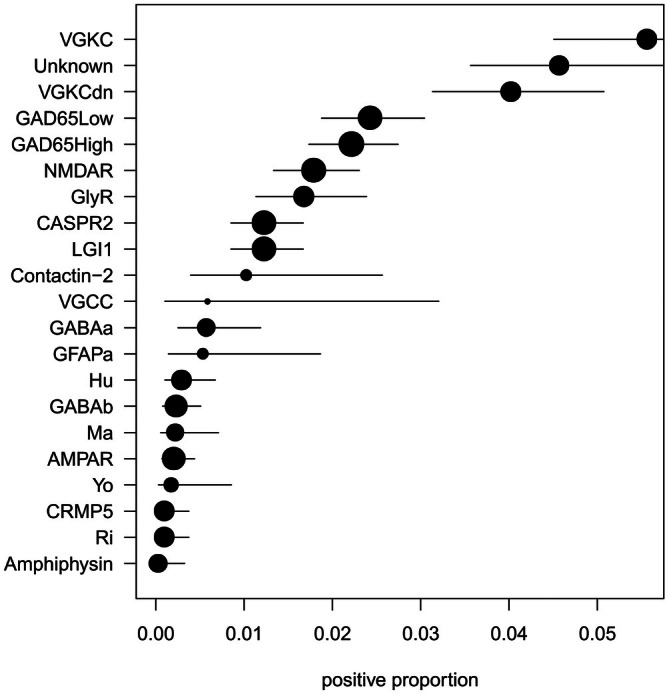
Neuronal autoantibody prevalence in epilepsy. Plots of autoantibody frequencyantibody target. Confidence intervals were estimated by DescTools. Data were drawn from Steriade et al.^21^

Unlike in AE, in which the immune response unquestionably drives seizures, this is less clear in autoimmune‐associated epilepsy. Autoimmune‐associated epilepsy is distinct from AE, in that patients have epilepsy in the context of detectable autoantibodies but in the absence of clear evidence of encephalitis. Estimates of the frequency of autoantibody detection in epilepsy vary widely between studies.^21^ This is at least in part because many of the autoantibodies detected in studies of autoimmune‐associated epilepsy are unlikely to be of direct immunopathogenic importance (Figure 1).^21^ For example, antibodies against voltage‐gated potassium channel complexes (VGKCCs) in which both LGI1 and CASPR2 antibodies are absent (called “double negative” VGKCC antibodies^22^, ^23^). Additionally, antibody‐independent mechanisms, such as T cells, are likely to be highly important in autoantibody‐associated epilepsy, particularly in GAD antibody‐associated epilepsy.^20^

### 3.1 Predicting autoantibody positivity in epilepsy

**FIGURE 2:**
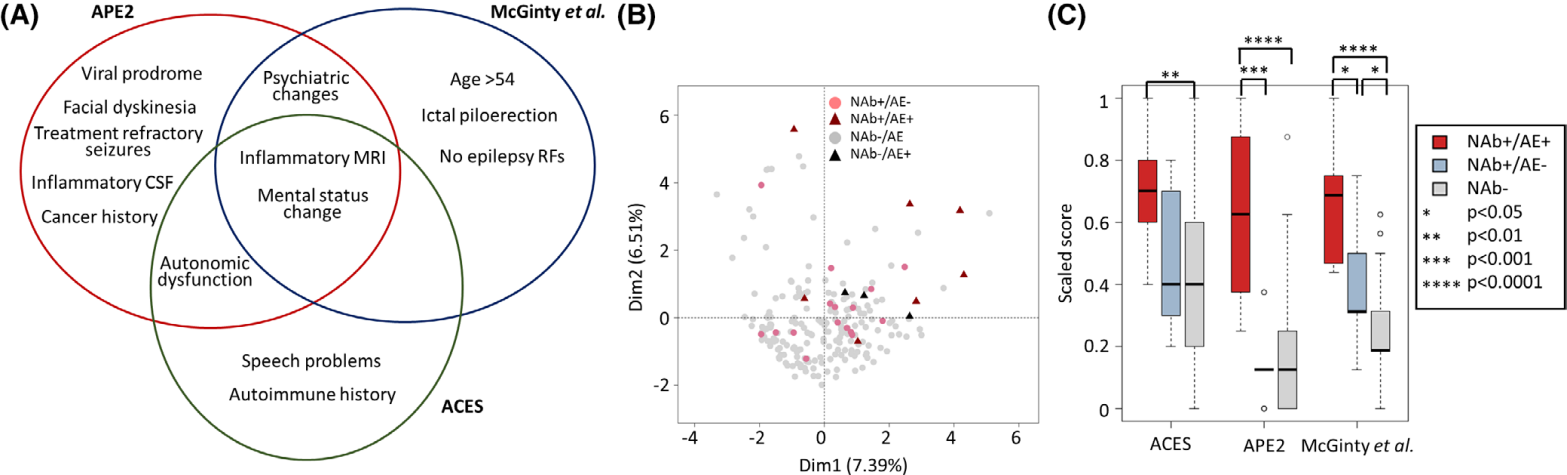
Scores to predict neuronal autoantibody (NAb) positivity in epilepsy. (A) A Venn diagram showing the areas of overlap between the different scoring systems.^15^, ^24^, ^26^ Note that some categories have been edited to emphasize commonalities. (B) A multiple factor analysis plot of patients with epilepsy from McGinty et al.^26^ NAb‐positive (pink) or NAb‐negative (gray) without autoimmune encephalitis (AE; dots), or NAb‐positive (red) with clinically diagnosed AE (triangles). (C) A boxplot of the scaled prediction scores calculated on the cohort from McGinty et al.^26^ stratified by NAb positivity and clinically diagnosed AE. Significance was calculated using the Wilcoxon rank‐sum test. ACES, antibodies contributing to focal epilepsy signs and symptoms score; APE2, Antibody Prevalence in Epilepsy and Encephalopathy score; CSF, cerebrospinal fluid; Dim1/2, multiple factor analysis dimensions 1 and 2; MRI, magnetic resonance imaging; RF, Reference.

Identifying individuals with epilepsy likely to test positive for autoantibodies has been a key focus of translational research. To this end, several groups have developed clinical scoring systems to identify patients with epilepsy, who have a high likelihood of testing positive for neuronal autoantibodies.^14^, ^24^, ^25^, ^26^ Despite being derived in different ways, all these scoring systems include consistent core features that predominantly capture an axis of AE clinical and paraclinical characteristics (Figure 2A).

In a recent cohort of prospectively recruited patients with new onset focal epilepsy, the major driver of clinical and paraclinical variation in the dataset was not the presence or absence of neuronal autoantibodies per se but rather whether the patient had been diagnosed with AE (Figure 2B).^26^ The scores were better at identifying AE rather than neuronal autoantibody positivity (Figure 2C). Furthermore, patients with epilepsy associated with neuronal surface antibodies unexpectedly had better long‐term outcomes if not treated with immunotherapy, suggesting that immunotherapy is not required in all patients and that the autoantibody detected may be unrelated to the pathogenesis of epilepsy in those patients. A score designed to predict outcomes following immunotherapy in this patient group, the RITE (Response to Immunotherapy in Epilepsy) score, demonstrated that clinical and paraclinical features of AE were highly predictive of outcome.^25^, ^27^

Overall, it is likely that the presence of autoantibodies alone does not imply causation in patients with epilepsy in the absence of clinical evidence of encephalitis. However, this conclusion is based on observational evidence and thus is vulnerable to multiple sources of potential bias. Therefore, the role of immunotherapy in autoimmune‐associated epilepsy should be assessed directly in a randomized controlled trial in an analogous manner to those testing immunotherapy in other oligosymptomatic presentations associated with autoantibody positivity. For example, the SINAPPS2 trial is currently assessing immunotherapy in the context of autoimmune psychosis.^28^

### 3.2 Beyond autoantibodies: T cells in autoimmune‐associated epilepsy

The presence of cytotoxic T cells within the temporal lobes of patients with GAD antibody‐associated epilepsy suggests that these are likely to be a key driver of disease pathogenesis in both GAD antibody‐associated epilepsy and autoimmune‐associated epilepsy more broadly.^29^ These T‐cell mechanisms are likely to be dynamic, with shifts in inflammatory transcript expression between early and late GAD antibody‐associated epilepsy.^29^ Although overall the response to immunotherapy in this group of patients is poor,^20^ some groups have suggested early immunotherapy may be beneficial.^30^ The role of T cells in autoimmune‐associated epilepsy is an area of ongoing research with clear translational potential.

## 4 AUTOIMMUNE MECHANISMS UNDERLYING ICTAL EVENTS: THE ROLE OF AUTOANTIBODIES

Autoantibodies have been identified in different subgroups of individuals with intractable epilepsy or encephalitis accompanied by seizures by the direct effect of autoantibodies on neuronal surface antigens (e.g., ion channels, receptor proteins) involved in synaptic transmission.^24^ Notably, some of these autoimmune antibodies have been demonstrated to cause significant pathological effects on neural cells both in vitro and in vivo.^31^, ^32^, ^33^ Passive transfer of autoantibodies derived from AE patients into the rodent brain provides insights into how autoimmunity can affect brain function, leading to alterations of neuronal excitability, seizures, impaired cognition, behavioral change, and other neurological and neuropsychiatric symptoms.^31^, ^32^, ^33^ Autoimmune antibodies can affect the induction or predisposition to seizures in distinct ways in different patients. For example, autoantibodies may directly or indirectly impair the levels, signaling, and function of glutamate (the major excitatory neurotransmitter in the central nervous system) or γ‐aminobutyric acid (GABA), or of their receptors, enzymes, ion channels, transporters, or other associated proteins.^34^, ^35^ It remains unknown to what extent autoantibodies contribute to seizure generation, and the precise effect of each newly discovered antibody remains largely unexplored.

**FIGURE 3:**
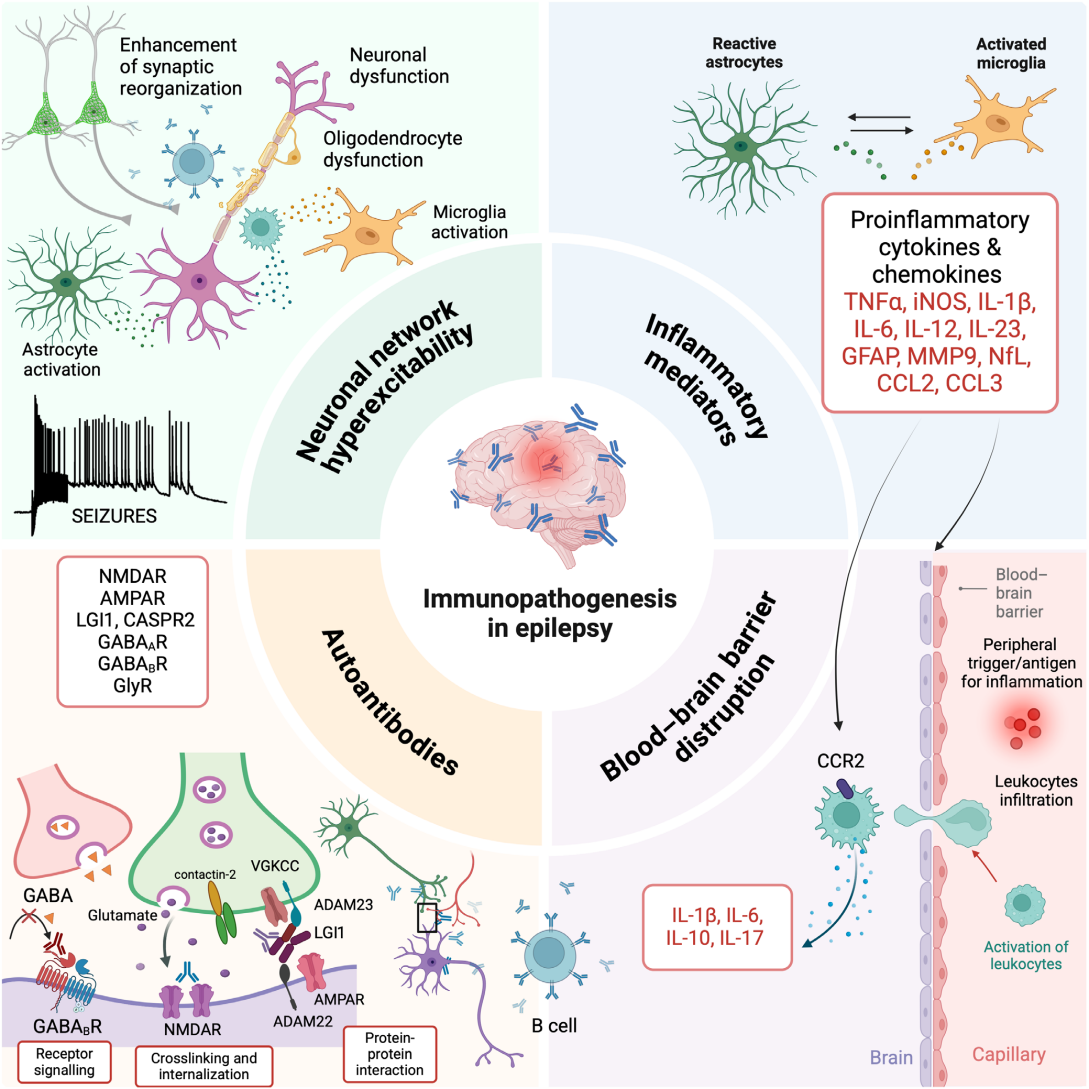
The involvement of immunity in the pathogenesis of epilepsy. Schematic representation shows the complex interactions between the immune system, central nervous system, inflammatory mediators, blood–brain barrier disruption, and autoantibodies, which contribute to neuronal network hyperexcitability in the pathogenesis of epilepsy. ADAM, a disintegrin and metalloproteinase domain‐containing protein; AMPAR, α‐amino‐3‐hydroxy‐5‐methyl‐4‐isoxazole propionic acid receptor; CASPR2, contactin‐associated protein‐like 2; CCL2, C‐C motif chemokine ligand 2 (also known as monocyte chemoattractant protein‐1, MCP‐1); CCL3, C‐C motif chemokine ligand 3 (also known as macrophage inflammatory protein‐1α, MIP‐1α); GABA, γ‐aminobutyric acid; GABA_A_R, GABA type A receptor; GABA_B_R, GABA type B receptor; GFAP, glial fibrillary acidic protein; GlyR, glycine receptor; IL, interleukin; iNOS, inducible nitric oxide synthase; LGI1, leucine‐rich glioma inactivated 1; MMP9, matrix metalloproteinase 9; NfL, neurofilament light chain; NMDAR, N‐methyl‐d‐aspartate receptor; TNFα, tumor necrosis factor α; VGKCC, voltage‐gated potassium channel complex. Created in bioRender (https://biorender.com).

Autoimmune‐mediated mechanisms can be broadly categorized into several modes of action, which are schematically illustrated in Figure 3. These include:

1. Effects on receptors, such as activation, inhibition, degradation, internalization, or structural modification: NMDAR, glycine receptors, GABA type B receptor, and α‐amino‐3‐hydroxy‐5‐methyl‐4‐isoxazole propionic acid glutamate receptor (AMPAR).
2. Alteration of enzymes and signaling molecules: GAD, CASPR2, LGI1, and dipeptidyl–peptidase‐like protein 6.
3. Promoting glial proliferation.

As these categories are visually summarized in the figure, a detailed mechanistic description is not provided here, but the listed examples illustrate the diversity of immune‐mediated effects on neuronal and glial targets.

Experimental studies conducted with antibodies from patients with NMDAR antibody encephalitis suggest that antibodies against NMDAR may contribute to epileptogenesis along with behavioral changes such as memory deficits.^31^, ^33^, ^36^ NMDAR antibodies decrease the expression of synaptic NMDAR expression on the cell surface by internalization along with reduced synaptic excitatory transmission and prominent changes in NMDAR‐mediated currents.^32^, ^36^ In several animal model studies, administration of NMDAR‐IgG obtained from the sera or cerebrospinal fluid of patients reduced the threshold for acute seizure induction rather than directly influencing the occurrence of spontaneous seizures.^31^, ^36^ This could result from an effect of autoantibodies against alternative antigenic targets beyond NMDAR that may be present in the patient‐derived total IgG pools administered to animals. In support of this argument, infusion of patient‐derived anti‐NMDAR monoclonal antibodies that interact exclusively with the target epitopes of the NMDAR can caused seizures (in the absence of behavioral symptoms) without any triggering agent.^37^ As discussed during this WONOEP session, the seizure outcome in antibody‐mediated animal models is determined by epitopes recognized by the IgG of individual patients, the animal species used (mouse vs. rat), and potentially also the method by which IgG is administered. In recent research, LGI1 antibodies were found to be pathogenic in a passive transfer mouse model in which patient‐ or control‐derived IgG was transferred into the cerebral ventricle.^38^ Mice infused with LGI1‐IgG exhibited memory impairment that partially reversed after stopping the infusion. Notably, no spontaneous seizures were observed in this model.^38^ However, LGI1‐IgG did lead to significant reductions in the density of both total and synaptic Kv1.1 potassium channels, and AMPAR clusters resulted from the interference of LGI1 interactions with presynaptic a disintegrin and metalloproteinase 23 (ADAM23) and postsynaptic ADAM22 proteins (Figure 3). This disruption also led to increased presynaptic excitability and glutamatergic transmission, as evidenced by in vitro experiments,^39^ but not sufficient to cause spontaneous seizures in vivo.

Building on this, the study by Upadhya et al. demonstrated that patient‐derived monoclonal LGI1 autoantibodies, when infused into rodents via intracerebroventricular administration, directly elicited both convulsive and nonconvulsive spontaneous seizures, alongside behavioral alterations and discernible brain magnetic resonance imaging changes.^40^ These findings underscore that LGI1 autoantibodies can actively provoke epileptogenesis and pathological neuronal activity in vivo, rather than merely altering synaptic function in a laboratory setting.

Based on these findings, autoantibodies derived from patients are pathogenic, and they may also cause impairments in spatial and recognition memory and promote astrocyte proliferation.^36^ We conclude that modeling patient‐derived autoantibodies in the rodent brain in vivo is an important strategy for elucidating these mechanisms. However, established in vivo models must meet the following criteria: (1) development of symptoms (modeling the molecular, synaptic, and behavioral effects of the specific autoantibody) in animals following the infusion of the patient‐derived IgG but not the control IgG obtained from healthy individuals; (2) determination of the concentration of patient‐derived autoantibodies transferred to the animal; (3) demonstration that the infused autoantibodies predominantly interact with the target antigen (e.g., NMDAR in the hippocampus) in the brain regions and are specifically recognized by these receptors; and (4) demonstration of a concentration‐dependent effect on the target antigen (such as decrease in receptor expression or exhibiting agonist or antagonist effects), with this effect being related to the duration of antibody administration.

## 5 INNATE AND ADAPTIVE IMMUNE MECHANISMS UNDERLYING ICTAL EVENTS: THE ROLE OF NEUROINFLAMMATION

Beyond autoimmune epilepsy, there is compelling evidence that inflammation, driven by both innate and adaptive immune responses, is likely to contribute to seizure occurrence, although the precise mechanisms remain largely unknown.^41^ It is most likely that the mechanisms responsible for the development of epilepsies and the generation of spontaneous recurrent seizures are multifactorial. Recent research has identified several new molecular mediators of neuroinflammation,^3^, ^42^ offering potential insights into pathogenic mechanisms and targets for antiseizure therapy. Soluble inflammatory mediators released by resident and circulating immune cells, as well as endothelial cells following blood–brain barrier (BBB) damage, have been a key focus (Figure 3). Additionally, the overproduction of free radicals due to reactive oxidative stress, and changes in mRNA expression of mediators related to inflammasome and proinflammatory pathways, have garnered significant attention.

### 5.1 Inflammatory soluble mediators as fingerprints of ictogenesis

Brain inflammation may either precede or follow seizures, and may arise secondarily to a primary systemic immune activation.^41^, ^43^ The established role of cytokines in triggering neuroinflammation, increasing neuronal excitability, and impairing the BBB, coupled with their recognized effect on ictogenesis in various epileptic disorders, has positioned these inflammatory mediators as crucial subjects in scientific investigation.^44^, ^45^ Inflammatory mediators released by both resident and circulating immune cells have been investigated for their role in affecting brain excitability and/or seizure threshold in AE. Seizures are common symptoms of AE,^46^ and although an epileptogenic potential has been shown for some antibodies against neuronal surface antigens, AE pathophysiology is complex, and antibody‐independent mechanisms likely contribute to seizures. Recent studies identified an altered systemic immune profile in the secretome of peripheral blood mononuclear cells (PBMCs) derived from AE patients compared to healthy subjects.^47^ Intriguingly, electrophysiological recordings revealed the capability of the secretome exclusively from PBMCs of AE patients to promote ictal events in a heterologous in vitro brain preparation. Morphological analysis and reconstruction of astrocytes and microglial cells also demonstrated brain parenchymal activation. The net effect of brain inflammation on neuronal network excitability likely depends on a combination of mediators and on the balance between proinflammatory cytokines or chemokines, and anti‐inflammatory mechanisms which, upon entering the brain parenchyma, contribute to induce ion channel and other receptor modifications that are permissive for neuronal hyperexcitability. Along with inflammatory cytokines, matrix metalloproteinase 9 (MMP9) and redox imbalance have been established to be involved in the immunopathogenesis of epilepsy by leading to reactive gliosis and increased IL‐1β and toll‐like receptor 4 expression.^48^ Accordingly, inhibition of MMP9, which degrades extracellular matrix around the vasculature, rescues these effects.^49^ High rates of oxidative metabolism coupled with increased inflammatory cytokines and MMP9 levels have been reported in pediatric epilepsy. Additionally, a positive correlation between these markers and seizure severity and frequency was identified.

### 5.2 Astrocytes and microglia as drivers of inflammatory signaling

Cytokines and related signaling molecules act as neuromodulators^50^ by activating their cognate receptors also on glial cells. This activation affects their function and modulates the circuits to which they contribute, leading to a reduction of seizure threshold. In turn, seizure activity per se promotes the synthesis of inflammatory mediators in parenchymal glial cells in brain regions involved in seizure generation, creating a vicious cycle. Accordingly, research is revealing how different glial cell types respond to and drive inflammatory signaling and seizures.

Glial fibrillary acidic protein (GFAP) is currently under investigation as a promising serum biomarker of reactive astrogliosis in various neurological disorders. Its potential diagnostic utility in epilepsy has also been explored. In a prospective cross‐sectional study, plasma GFAP was analyzed using SIMOA technology in adult patients diagnosed with epilepsy, psychogenic nonepileptic seizures (PNES), or other nonepileptic conditions. The results showed that GFAP levels were significantly higher in patients with epilepsy compared to those with PNES.^51^

Neuroinflammation plays a key role in the pathogenesis of epilepsy by driving sustained microglial activation and subsequent neurodegeneration.^3^ Microglia represent one of the major sources of proinflammatory molecules and play a critical role in determining the spatial and temporal extent of inflammation.^52^, ^53^ Microglial cells respond to the epileptic environment and change their activity of releasing proinflammatory and anti‐inflammatory cytokines^54^ by phagocytosing apoptotic and living cells,^55^ by engulfing synapses,^56^ and by stripping synapses.^57^ The role of microglial proliferation and activation in the development of acquired epilepsy is still unknown; investigation into this issue involved examining the effects of microglial depletion immediately following status epilepticus in a mesial temporal lobe epilepsy mouse model. Microglia depletion significantly downregulated the expression of proinflammatory cytokines but did not lead to a significant reduction in seizure frequency.^58^

### 5.3 Inflammatory gene expression analysis: A novel investigation approach

To assess the role of neuroinflammation, particularly inflammasome and innate immune cell pathways, in drug‐resistant temporal lobe epilepsy (DRTLE), the expression of various neuroinflammatory genes in temporal lobe samples from DRTLE patients has been investigated and quantified. Several genes related to inflammasome and proinflammatory pathways, such as *NLRP3*, *CASP1*, *TNF*, *IL1B*, *IL18*, *CCL2*, *CCL3*, and *CXCL9*, as well as genes encoding monocyte/macrophage/microglia markers (*TMEM119*, *CD14*, *CD68*), were found to be upregulated in DRTLE patients compared to postmortem controls without neurological disease. This finding suggests a possible role of innate immunity in DRTLE pathogenesis.^59^

### 5.4 *Toxoplasma gondii* infection: A risk factor for epilepsy

**FIGURE 4:**
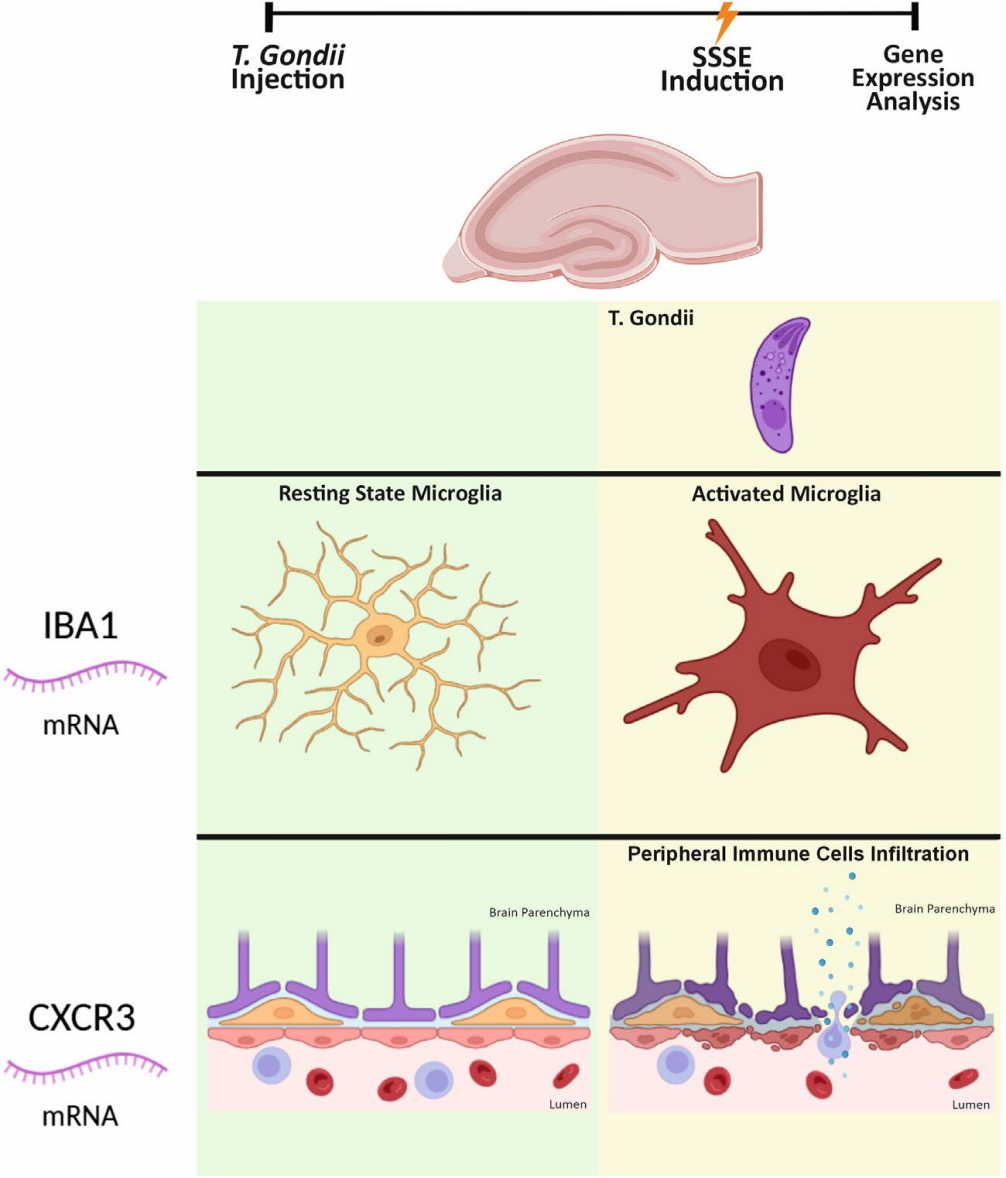
Preexisting *Toxoplasma gondii* infection involvement in epileptogenesis. Chronic *T. gondii* infection can modify the neuroinflammatory environment and influence epileptogenic processes in males in the long term and promote spontaneous recurrent seizures in sham *T. gondii* mice. CXCR3, CXC motif chemokine receptor 3; IBA1, ionized calcium‐binding adapter molecule 1; SSSE, self‐sustained status epilepticus.

Chronic low‐grade neuroinflammation resulting from infectious diseases can increase the risk of epilepsy by altering the brain's inflammatory environment, neural connectivity, and overall structure.^60^ The parasite *Toxoplasma gondii* (*T. gondii*), which infects approximately one third of the world's population, is a potential, often overlooked, and preventable risk factor for epilepsy.^61^ Studies have investigated whether preexisting *T. gondii* infection and the resultant host immune response may shape the neuroinflammatory environment and facilitate the development of epilepsy.^62^ Experimental results showed that *T. gondii*‐infected mice developed spontaneous seizures and inflammatory gene expression changes, both indicative of an exacerbated neuroimmune response and oxidative stress. These data strongly support the notion that preexisting *T. gondii* infection alters the neuroinflammatory environment, making the brain more susceptible to developing epilepsy (Figure 4).^63^

## 6 CONCLUSIONS AND FUTURE PERSPECTIVES

WONOEP XVII focused on the extracellular space in epilepsy, and this appraisal summarizes the content and discussions explored in the session on the immunopathogenesis of epilepsy, bridging clinical, epidemiological, and neurophysiological insights. We highlight the role of neuron‐specific autoantibodies in triggering seizures and investigated inflammation‐related biomarkers as indicators of epilepsy's cause and progression. This exploration clearly demonstrated that neuroinflammation plays a significant role in epilepsy, connecting both the innate and adaptive branches of the immune system. Collectively, these neuroimmune interactions represent an area of great opportunity to understand the causes of epilepsy and, more importantly, to develop innovative treatments. However, further research is crucial to fully understand how autoantibodies and neuroinflammatory changes associated with epilepsy lead to seizures in individual patients. As highlighted in the audience discussion following this session, it is time to consider how to best test potential immune‐directed treatments through randomized, controlled trials. Undoubtedly, this will require national and international collaboration, a challenge the academic and clinical epilepsy community is well prepared to meet.

## FUNDING INFORMATION

The work of N.Ç. is supported by Health Institutes of Türkiye Group B R&D call (grant #35809) and the Scientific Research Projects Coordination Unit of Istanbul University (grant #40486). D.C.H. acknowledges research support from a research grant (16/RC/3948 and 21/RC/10294_P2, FutureNeuro). A.S.G. received research funding from NINDS NS127524, the US Department of Defense (W81XWH‐22‐1‐0210, W81XWH‐22‐1‐0510, EP220067, HT9425‐24‐1‐0134), the Isabelle Rapin and Harold Oaklander Child Neurology Research Fund in the Isabelle Rapin Child Neurology Division, and the Abbe Goldstein/Joshua Lurie and Laurie Marsh/Dan Levitz families. A.E.H. is supported by the National Institute for Health Research (NIHR) Oxford Health Biomedical Research Centre, the Medical Research Council (UK; MR/X022013/1), UCB‐Pharma, and MyAware. L.L. is supported by the Italian Health Ministry (RF‐2021 12372526) and Fondazione Lega Italiana Contro l’Epilessia grant 2024. The views expressed are those of the authors and not necessarily those of the NHS, the NIHR, or the Department of Health. P.M.C.‐E. is supported by the SAID (FLPF24‐0237761657), the Medical Research Future Fund stem cell therapy missions grant (MRF1201781), and the US Department of Defense Epilepsy Research Program (DoD ERP IDA, grant #EP200022, DoD ERPA RPA, grant #EP220067).

## CONFLICT OF INTEREST STATEMENT

A.E.H. has served on an advisory board for Argenx. A.S.G. is a consultant for Synergy Medical Solutions and has received royalties for publications or editorial roles from Elsevier, Medlink, Wolters Kluwer, and Oxford. None of the other authors has any conflict of interest to disclose. We confirm that we have read the Journal's position on issues involved in ethical publication and affirm that this report is consistent with those guidelines.
